# Targeted Metabolomics Shows That the Level of Glutamine, Kynurenine, Acyl-Carnitines and Lysophosphatidylcholines Is Significantly Increased in the Aqueous Humor of Glaucoma Patients

**DOI:** 10.3389/fmed.2022.935084

**Published:** 2022-07-22

**Authors:** Alejandro Lillo, Silvia Marin, Joan Serrano-Marín, Nicolas Binetti, Gemma Navarro, Marta Cascante, Juan Sánchez-Navés, Rafael Franco

**Affiliations:** ^1^CiberNed, Network Center for Neurodegenerative Diseases, National Spanish Health Institute Carlos III, Madrid, Spain; ^2^Department of Biochemistry and Physiology, School of Pharmacy and Food Science, Universitat de Barcelona, Barcelona, Spain; ^3^Department of Biochemistry and Molecular Biomedicine, Faculty of Biology, Universitat de Barcelona (UB), Barcelona, Spain; ^4^Institute of Biomedicine of University of Barcelona (IBUB), University of Barcelona (UB), Barcelona, Spain; ^5^CIBEREHD, Network Center for Hepatic and Digestive Diseases, National Spanish Health Institute Carlos III (ISCIII), Madrid, Spain; ^6^Molecular Neurobiology Laboratory, Department of Biochemistry and Molecular Biomedicine, Universitat de Barcelona, Barcelona, Spain; ^7^Department of Ophtalmology, Oftalmedic and I.P.O. Institute of Ophthalmology, Palma de Mallorca, Spain; ^8^School of Chemistry, Universitat de Barcelona, Barcelona, Spain

**Keywords:** lipidomics, glutamine, eye disease, mass spectrometry, biomarker, glaucoma, kynurenine, mitochondria

## Abstract

The composition of the aqueous humor of patients with glaucoma is relevant to understand the underlying causes of the pathology. Information on the concentration of metabolites and small molecules in the aqueous humor of healthy subjects is limited. Among the causes of the limitations is the lack of healthy controls since, until recently, they were not surgically intervened; therefore, the aqueous humor of patients operated for cataract was used as a reference. Sixteen aqueous humor samples from healthy subjects undergoing refractive surgery and eight samples from glaucoma patients were used to assess the concentration of 188 compounds using chromatography and mass spectrometry. The concentration of 80 of the 188 was found to be reliable, allowing comparison of data from the two groups (glaucoma and control). The pattern found in the controls is similar to, but not the same as, that reported using samples from “controls” undergoing cataract surgery. Comparing data from glaucoma patients and healthy subjects, 57 of the 80 compounds were significantly (*p* < 0.05) altered in the aqueous humor. Kynurenine and glutamine, but not glutamate, were significantly increased in the glaucoma samples. Furthermore, 10 compounds were selected considering a statistical score of *p* < 0.0001 and the degree of change of more than double or less than half. The level of C10 (decanoyl)-carnitine decreased, while the concentration of spermidine and various acyl-carnitines and lysophosphatidylcholines increased in glaucoma. Principal component analysis showed complete segregation of controls and cases using the data for the 10 selected compounds. The receiver operating characteristic curve these 10 compounds and for glutamine allowed finding cut-off values and significant sensitivity and specificity scores. The concentration of small metabolites in the aqueous humor of glaucoma patients is altered even when they take medication and are well controlled. The imbalance affects membrane components, especially those of the mitochondria, suggesting that mitochondrial abnormalities are a cause or consequence of glaucoma. The increase in glutamine in glaucoma is also relevant because it could be a means of keeping the concentration of glutamate under control, thus avoiding its potential to induce the death of neurons and retinal cells. Equally notable was the increase in kynurenine, which is essential in the metabolism of nicotine adenine dinucleotides.

## Introduction

The aqueous humor (AH) is necessary for correct visual perception. Among others, the aqueous humor maintains the three-dimensional structure of the mammalian eye and facilitates the focusing of images on the retina. Consequently, it must be transparent and have an appropriate refractive index. The first report on the physiology of AH formation and chemical composition was made about a century ago by Yudkin (1). Although it was supposed to have few components to achieve transparency, various studies have found molecules as diverse as vitamins, amino acids, intermediate metabolites, and lipids (2–4). Unfortunately, AH is a bodily fluid that requires surgery to obtain a sample, which limits its use in the diagnosis and/or prevention of eye diseases. The composition of the AH is likely to be altered in diseases of the eye.

One of the most prevalent eye diseases, glaucoma, is the second leading cause of permanent blindness worldwide. Although there are several therapeutic approaches, including pharmacological options, trabeculoplasty (application of a laser beam to the trabecular meshwork to increase AH outflow), and surgical options, the etiology and pathophysiology remain unclear (5). In poor/developing countries where medication is scarce and/or unaffordable, glaucoma becomes a more serious problem (6). The main alteration is the accumulation of AH inside the eye, thus causing an increase in intraocular pressure (IOP) that leads to retinal damage and, in the long run, alterations in the optic nerve (7). AH, which comes mainly from the stromal fluid of the ciliary processes, is secreted in the posterior chamber of the anterior cavity of the eye (8). The abnormal accumulation of fluid in glaucoma is mainly due to poor drainage through the trabecular meshwork and/or increased AH production (7). Outflow can be affected for a number of reasons, including disturbances that increase drain resistance. For example, it has been described that endothelial cells that have a greater rigidity in the cytoskeleton can inhibit the formation of pores that facilitate the exit of AH (9). Another factor is the decrease in the volume of Schlemm's canal and/or the surface of the collecting canals (10). In addition, the activation of retinal microglia directly affects the physiology of the trabecular meshwork (11), which leads to a deregulation of the outflow of AH. It is hypothesized that the composition of AH may help understand the causes of glaucoma and ultimately improve therapeutic interventions.

Glaucoma, as well as other diseases that course with inflammation and neurodegeneration, e.g., Alzheimer's disease and Parkinson's disease, have been linked to mitochondrial deterioration (12, 13). On the one hand, one of the most common mechanisms of neuronal death is excitotoxicity caused by the increase in extracellular glutamate (14–16); therefore, it is expected that altered glutamate metabolism and/or glutamate-related events may be affecting the risk of glaucoma. In this sense, it would be relevant to know the level of glutamate and glutamine in AH in healthy controls and in glaucoma patients. On the other hand, inflammatory events in the retina (11) are accompanied by altered metabolic dysfunction, oxidative stress, and altered lipid metabolism. These processes that occur in the mitochondria affect the composition of the surrounding extracellular medium. Until recently, the quantification of some metabolites likely to be altered in inflammation has been difficult for technical reasons. An example is the acyl-carnitines whose variation in the retina can be translated into AH through microfluidic circulation (8). Therefore, finding changes in AH composition related to mitochondrial function could be a key step in advancing our understanding of the pathophysiological mechanisms underlying glaucoma. The opinion that the inflammatory component is important for the development of glaucoma is growing (12, 13).

The composition of the AH of patients with open-angle glaucoma, compared with that of patients with a variety of eye diseases other than glaucoma, shows increased levels of creatinine and decreased levels of taurine and spermine. The same metabolomics study led to the identification of a further 12 compounds when *p*-values were thresholded at 0.05 (3). In a more recent conceptually similar study using a different methodological platform, 22 metabolites were found whose levels were altered in AH samples from patients with open-angle glaucoma (17); control samples in this study were obtained from people undergoing cataract surgery. The compounds with higher score were cyclic AMP (cAMP), 2-methylbenzoic acid, 3'-sialyllactose, lysophosphatidylcholines 18:0 and 15:0 and hypoxanthine. The fold change for these compounds was moderate and more marked (2/3-fold) for uric acid, hydroxyphenyllactic acid N^6^-succinyl adenosine. Using healthy controls a lipidomic-based showed that 37 of 110 lipids were significantly altered in samples from glaucoma patients, the majority of the 37 were increased in glaucoma with the exception of some lysophosphatidylcholines (18). In the present paper we compared the composition of AH from open-angle glaucoma patients and healthy controls using a targeted metabolomics platform that determines the concentration of 188 metabolites, amino acids, biogenic amines, lysophosphatidylcholines, acyl-carnitines, sphingomyelins and total sugars. The controls in this work did not suffer from cataracts or any other eye disease.

## Methods

### Subjects

A total of 23 samples were collected, 16 were from healthy controls; 5 were men and 9 women, mean age was 36 (range 25–56), and 8 were from open angle glaucoma patients; 4 were men and 4 women, mean age was 67.5 (range 51–81). None of the individuals had previously undergone eye surgery. Comorbidities in the patient's group were hypertension (*n* = 5), hyperlipidemia (*n* = 3) and altered thyroid function (*n* = 1). Participants were informed and, in terms of ethical standards, this study adhered to the tenets of the declaration of Helsinki. The study has been evaluated by the *Comitè d'Ètica de la Investigació de les Illes Balears (CEI-IB)* and approved under the conditions of good practices of the *Illes Balears* Health Service in the use of information systems and in the treatment of personal data (document available at: (https://www.ibsalut.es/docs/docs/CA/cbp_cat.pdf; accessed on June 3, 2022).

All glaucoma patients were well controlled with topical drop therapy. AH was collected at the beginning of the intervention; both in the glaucoma group and in the control group there were individuals with myopia or hyperopia but without cataracts or ocular pathologies. The ocular pressure of controls and patients was within the reference interval. The mean of controls and patients was −4.5 diopters (range −2.5 to −7 diopters). The controls were not diagnosed with any pathology. No previous surgery is reported. Individuals underwent refractive lensectomy or collamer lens (ICL) implantation (without removing the natural eye lens). All surgeries were performed by the same surgeon (J.S.N.) on an empty stomach, and after pupil dilation and disinfection with 5% ophthalmic povidone-iodine, under topical anesthesia and identical preoperative and operative protocol. The first side port was made about 1 mm width under the microscope in the operating room, and aspirate 100-150 μl with a 27G needle to avoid deepening into the anterior chamber of the eye. Each sample was transferred to a 0.5 mL Eppendorf tube and stored at −80 C until analysis.

### Metabolomics

The approach in the paper was selected to determine as many metabolites as possible also aiming at the determination of compounds whose level is difficult to assess due to technical issues, for instance the measurement of carnitines having acyl chains of different number of carbons. Accordingly, targeted metabolomics using the AbsoluteIDQ™ p180 Kit (Biocrates Life Sciences, Innsbruck, Austria), was performed. This approach allows the absolute quantitation of up to 188 metabolites, including amino acids, biogenic amines, hexoses, acyl-carnitines, phospho- and sphingolipids. Individual metabolites may be found in www.biocrates.com/products/research-products/absoluteidq-p180-kit. Up to 30 μl of AH was plated in each well. Standards, quality controls and internal standards were placed in the plate, which was processed according to manufacturer instructions. Derivatized samples were analyzed in the AB Sciex 6500 QTRAP MS/MS mass spectrometer (AB Sciex LLC, Framinghan, MA, USA) coupled to an Agilent 1290 Infinity UHPLC system (Agilent, Santa Clara, CA, USA), following the instructions provided with the kit. Spermine could not be detected in our hands, neither in standards nor in samples (values were below detection limit and/or too low in comparison to the lowest value of the standard). Analyst and the MetIDQ™ software packages were used to analyze the obtained data and calculate metabolite concentrations. Final concentrations were calculated taking into account the volume that was placed in each well.

### Statistical Analysis

Univariate analysis of data was performed using one-way ANOVA comparing one by one the 80 compounds whose concentration was reliably determined by MS. Significant differences were considered when *p* < 0.05. We selected those metabolites that meet both criteria: log_2_ fold change (FC) > |1| and *p* < 0.0001. The receiver operating curve (ROC) was constructed using IBM SPSS software. The Principal Component Analysis (PCA) was done using the R software.

## Results

### Summary of Findings in Samples From Healthy Controls Without a Diagnosis of Eye Disease

Analysis of data from healthy controls and patients diagnosed with glaucoma led to finding reliable concentrations for 80 compounds (see values of compounds in all samples in Supplementary Table S1). Metabolites with concentration values below the detection limit or whose values are not within the standard curve were not included in the analysis. We then compared our control data with the control data in the Buisset et al. (3) report, in which the same kit was used (3). Unlike the controls in our study, the controls in Buisset et al. study had cataracts. Table 1 shows the comparison of concentrations of the 22 compounds selected in the study by Buisset et al. (3) report [see Table 2 in (3)]. The concentrations of 23 compounds vary by more than two-fold or less than one-half (i.e., log_2_ fold change between −1 and 1). In our study, the concentration of taurine, butyrylcarnitine and glycine was lower by, respectively, 2.2, 2.3 and 3.3 times. Although the trend was to find lower values in our study, this was not always the case, for example, for acetylornithine and leucine among the compounds listed in Table 1.

**Table 1:** Comparison of concentration of metabolites (median) in controls with those in the Buisset et al. (3) report.

**Metabolite**	**Median in (3) (μM)**	**Median in the present report (μM)**
Creatinine	38.0	24.0
Propionylcarnitine (C3)	0.33	0.19
PC aa C34:1	0.10	0.10
Glutamine	580.0	563.3
Acetylcarnitine (C2)	3.05	1.81
Taurine	41.20	18.33
PC aa C36:2	0.045	0.034
PC aa C36:4	0.030	0.019
PC aa C38:4	0.037	0.019
SM C18:1	0.009	0.0065
Carnitine (C0)	14.8	9.32
PC aa C32:1	0.016	0.014
PC aa C34:2	0.056	0.056
Trans-4-OH-Proline	3.46	2.08
Isoleucine	74.6	73.7
PC aa C30:2	0.002	0.002
Alanine	251.0	159.3
Acetylornithine	0.16	0.34
Butyrylcarnitine (C4)	0.15	0.054
Lyso PC a C28:1	0.018	0.0098
Leucine	96.9	134.7
Glycine	21.95	6.60

*Only the data for compounds selected in the Buisset et al. (3) report are shown. Compounds for which the ratio between both values is higher than 2 or lower than a half are in red*.

**Table 2:** Values of sensitivity, specificity and area under the curve obtained from the ROC plots in Figure 2.

**Metabolite**	**Sensitivity**	**Specificity**	**Cut-off (μM)**	**AUC^a^**
C2	1	0.937	2.58	0.992
C3	1	0.875	0.270	0.969
C3-DC (C4-OH)	1	0.937	0.012	0.945
C4	0.875	0.937	0.0943	0.945
C5	0.75	1	0.102	0.941
C10	0.875	1	0.084	0.984
PC aa C42:6	1	1	0.0582	1
PC ae C30:1	0.875	0.937	0.0093	0.914
PC ae C36:3	0.75	1	0.0112	0.938
PC ae C40:2	0.75	1	0.0058	0.914
Glutamine	0.875	0.812	623	0.805

a *AUC, Area under the curve*.

### Concentration of Total Sugars, Amino Acids and Biogenic Amines in the Aqueous Humor of Control Individuals

The methodology used only allows the determination of total sugars and the results show that it is high (>3 mM) and similar in control samples and in glaucoma samples (details in Supplementary Table S1).

The concentration of the 20 L-amino acids that can be present in proteins varies by 2 orders of magnitude. The lowest concentration was found for aspartic acid (1.5 μM) while the highest was for glutamine (550 μM). The glutamic acid (glutamate) level was (6.6 μM). In addition to glutamine, the amino acids whose concentrations exceeded 100 μM were: alanine, leucine, lysine, threonine and valine. In summary, the level of amino acids in AH was very heterogeneous and did not present any specific pattern (details in Supplementary Table S1).

Regarding biogenic amines, the highest levels were found for creatine (26.7 μM), taurine (17.3 μM) and methionine sulfoxide (14.5 μM), and spermidine was among the lowest (0.3 μM). Interestingly, 3,4-dihydroxyphenylalanine (DOPA), the precursor of catecholamines was detectable (0.14 μM). Kynurenine, which was significantly increased in glaucoma samples (see below), was also detectable (0.49 μM) (details in Supplementary Table S1).

### Concentration of Acyl-Carnitines in the Aqueous Humor of Control Individuals

The concentration of up to 13 carnitines in the AH of both control individuals and glaucoma patients was reliably determined. Information on these compounds is relevant since acyl-carnitines are involved in lipid transport across membranes. By far, carnitines C0, C2 and C3 were the most abundant with concentration values of 9.8, 1.7 and 0.2 μM, respectively. Other acyl-carnitines were at concentrations one to two orders of magnitude lower (Supplementary Table S1). Virtually all acyl-carnitine levels were altered in the glaucoma samples (see below).

### Concentration of Glycerophospholipids and Sphingomyelins in the Aqueous Humor of Control Individuals

Consistent with the hydrophobicity of glycerophospholipids and sphingomyelins and the high-water content of AH, the concentration of these compounds in this body fluid was relatively low. However, it was possible to reliably determine the concentration of 26 glycerophospholipids and 6 sphingomyelins. Sphingomyelin levels ranged from 0.04 μM (sphingomyelin C16:0) to 0.0021 μM (sphingomyelin C22:3) (Supplementary Table S1). Regarding glycerophospholipids, their levels ranged between 0.14 μM (C34:1 phosphatidylcholine) and 0.0025 μM (C40:2 phosphatidylcholine). Surprisingly, except for glycerophospholipid C36:6 and sphingomyelin SM C22:3, the levels of all detectable glycerophospholipids and sphingomyelins were significantly altered in glaucoma samples (Supplementary Table S1).

### Compounds Whose Concentration Is Altered in the AH of Glaucoma Patients

**Figure 1:**
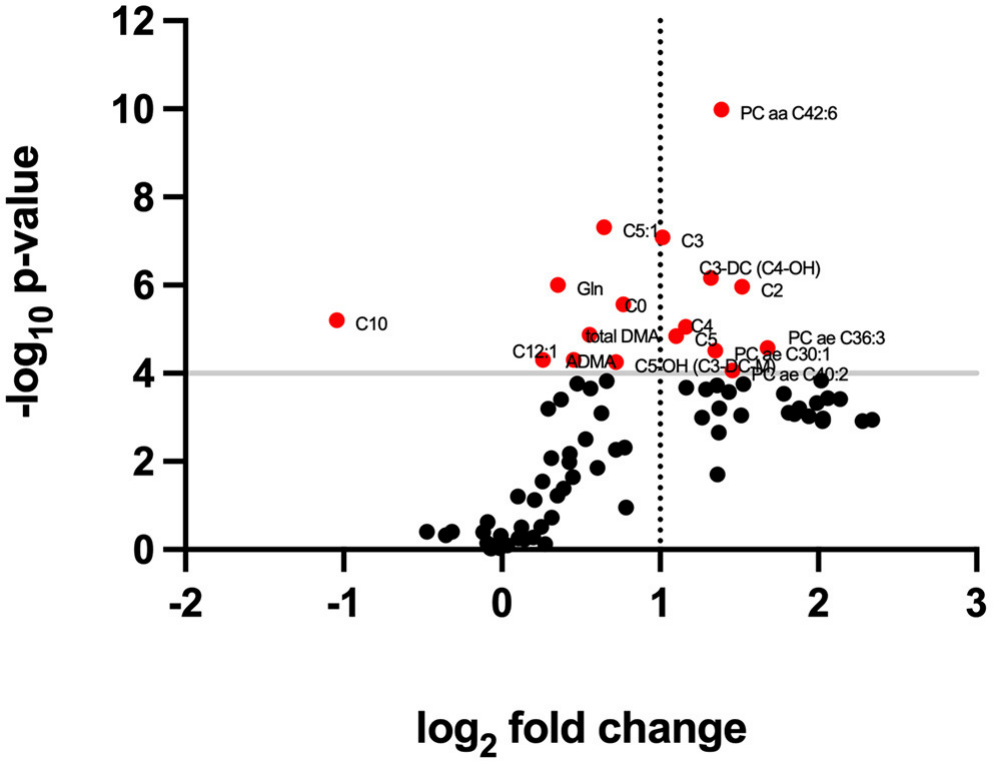
Volcano plot relating significance levels and fold change in data from glaucoma and control samples. Compounds for which differences have a *p* < 0.0001 score are shown in red.

**Figure 2:**
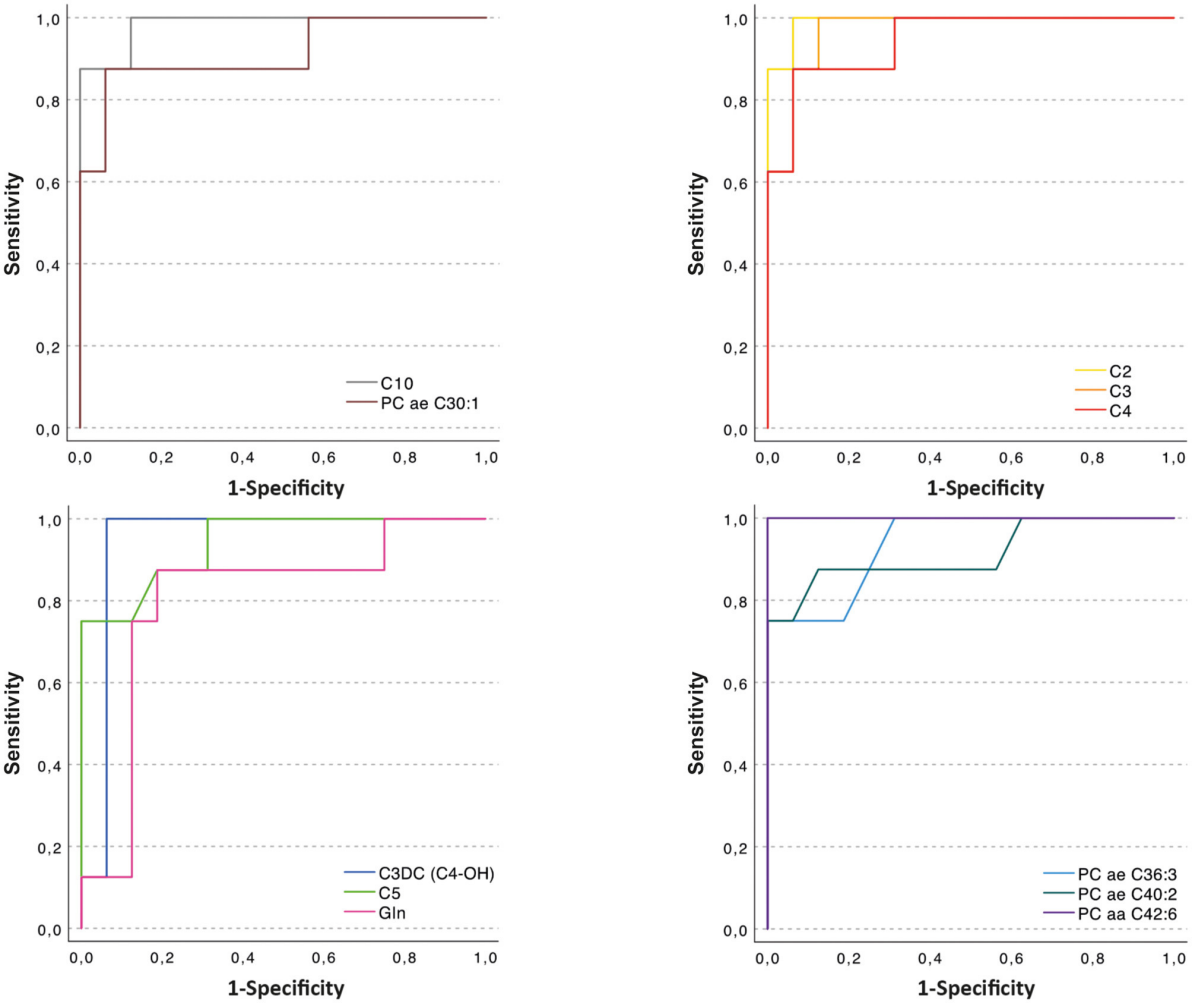
Sensitivity vs. specificity ROC curve of glutamine (Gln) and of the 10 compounds that have a *p* < 0.0001 score and whose log_2_ FC > |1|.

**Figure 3:**
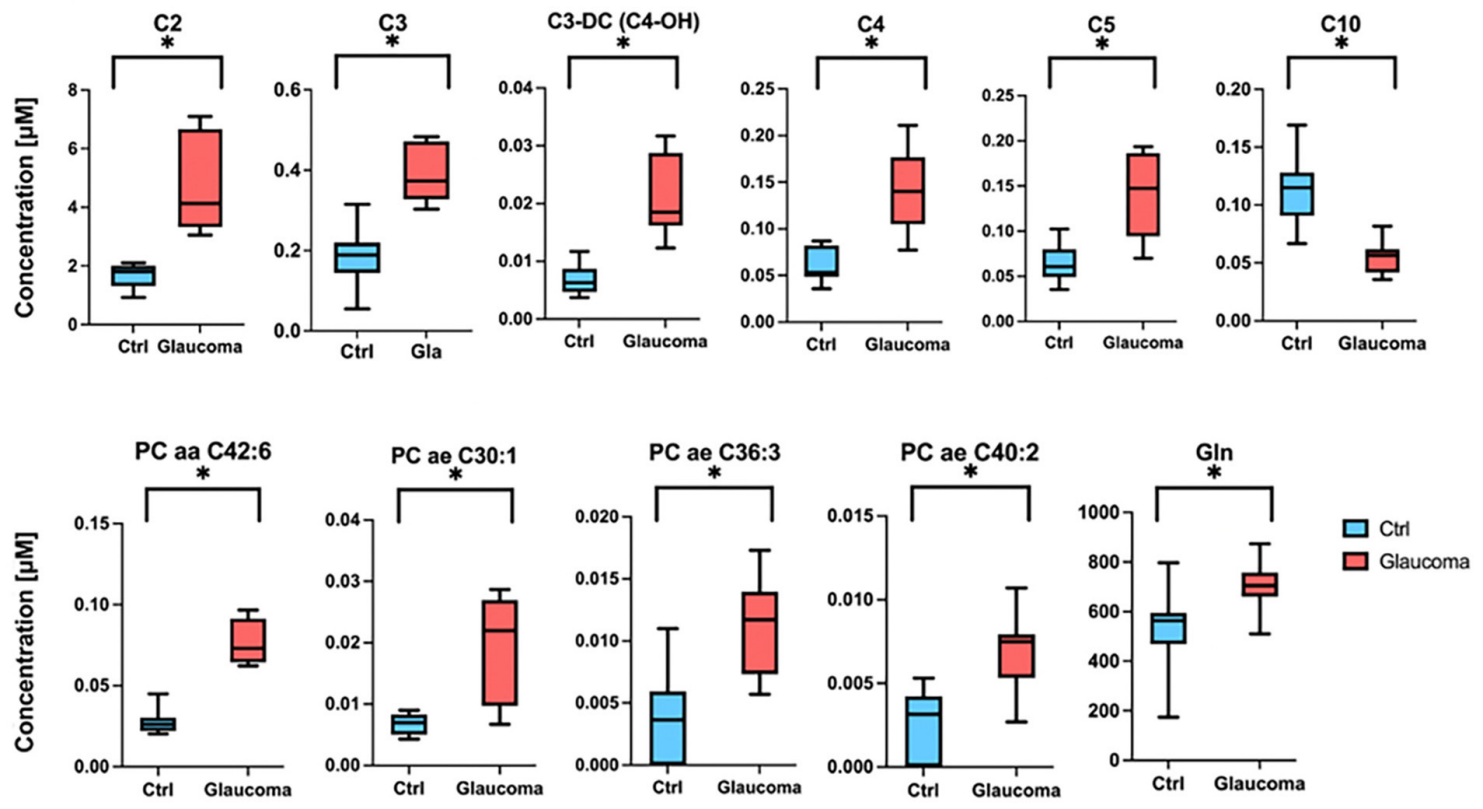
Concentration of glutamine (Gln) and of acyl-carnitines and lysophosphatidylcholines in aqueous humor (AH) from healthy individuals and glaucoma patients. Data are shown as median in box-and-whisker plots (whiskers denote the highest and lowest value determined for each compound in each group, control or glaucoma). *p < 0.05.

**Figure 4:**
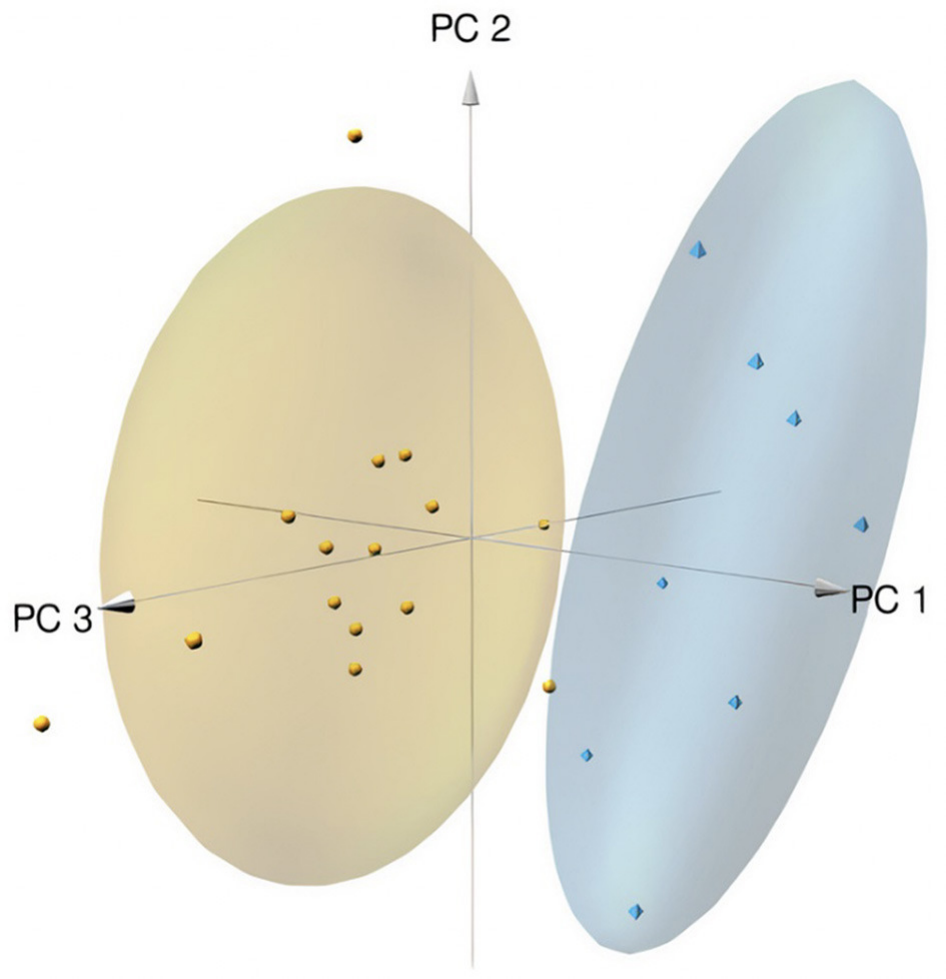
Principal component analysis graphic. The space and data for controls are in yellow and the space and data for glaucoma individuals are in blue. The principal component (PC) 1, the PC2, and the PC3 explains the 62.61%, the 20.31%, and the 11.20% of the variance between groups, respectively. See Supplementary Tables S2, S3 for details.

**Figure 5:**
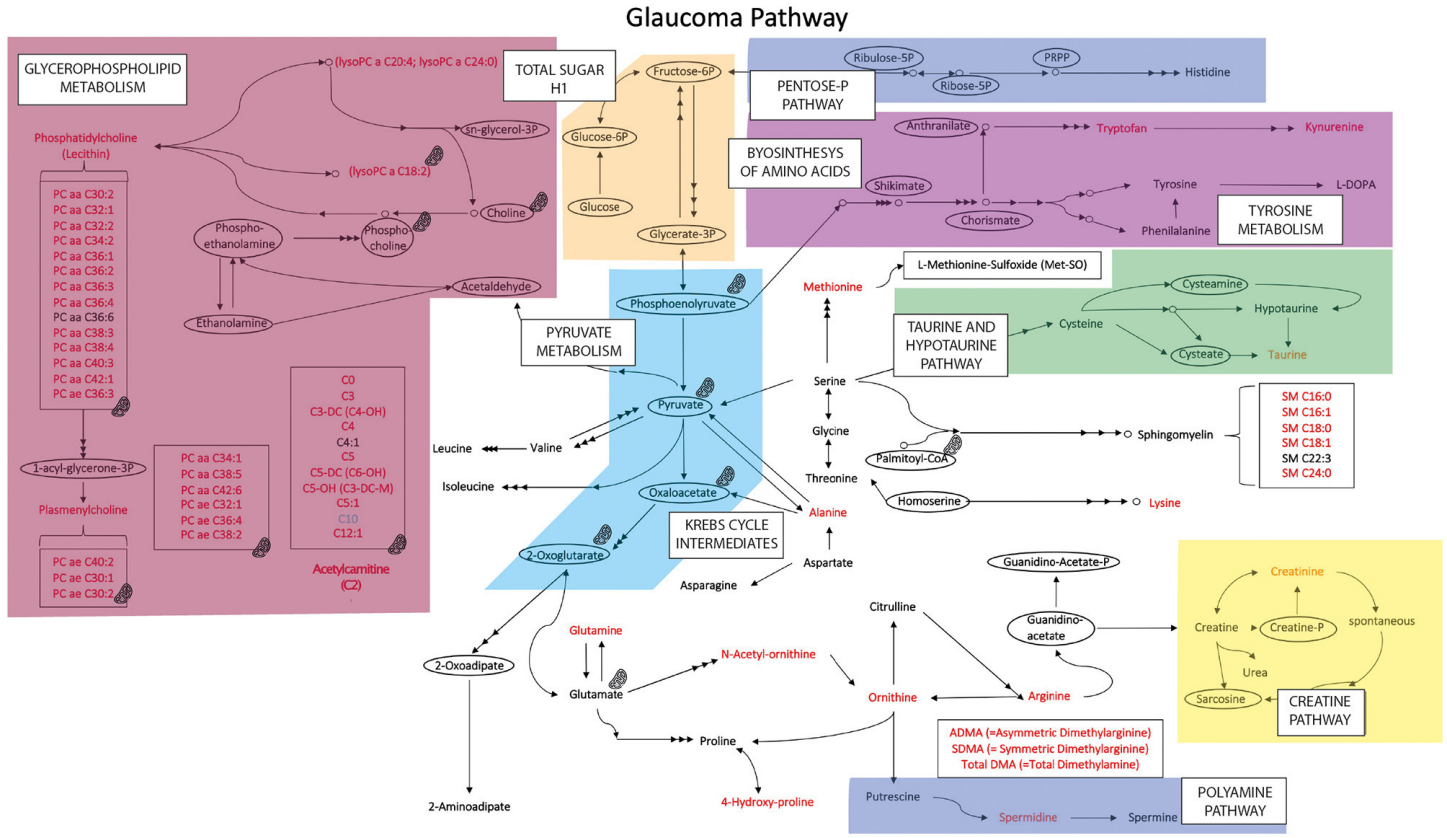
Metabolic pathway map summarizing the results obtained in glaucoma samples. In red the metabolites whose concentration is increased in the glaucoma samples (compared with values in samples from healthy controls), in light blue the metabolite whose concentration is reduced in glaucoma samples. Compounds within ellipses were not determined in this study; they are included as nodes in metabolism interconnections. Areas in color represent main metabolic pathways. 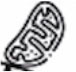 Icon indicates compound/group of compounds that may be in mitochondria or are directly/indirectly related to mitochondria.

When comparing data in samples from glaucoma patients and healthy controls, the concentration of 57 of 80 compounds was significantly different (indicated in red columns in Supplementary Table S1. A volcano plot (Figure 1) led to selecting the compounds whose range of variation between individuals with glaucoma and control was greater and with greater reliability (lower *p* value). A receiver operating characteristic (ROC) curve was constructed for the 10 compounds selected considering *p* < 0.0001 and log_2_ fold change (FC) > |1|; ROC for glutamine was also included because, in absolute values, the increase in glaucoma patients was marked (Figure 2). The area under the ROC (AUC) for all 11 compounds was close to 1 and the sensitivity and specificity were also close to one. Consequently, the cut-off values in Table 2 seem very reliable. Consistent with the modest fold change of glutamine concentration in glaucoma vs. control, the scores were the lowest among the 11 selected compounds; Sensitivity, specificity, and AUC values for glutamine were 0.875, 0.812, and 0.805, respectively. Glutamine concentration of AH is relatively high and the cut off value of this compound in AH from glaucoma patients is 623 μM (Table 2). Significant differences between controls and glaucoma patients are shown in Figure 3 for glutamine and the other 10 compounds. Principal component analysis shows good separation between controls and cases; Figure 4 shows a three-dimensional view of the Cartesian space where 75% of the individuals of each population would be located. The principal component (PC) 1, the PC2, and the PC3 explain, respectively, the 71.9%, the 7.9%, and the 5.3% of the variance between groups. To understand the level of general affectation within a biochemical framework, Figure 5 shows the compounds that are differentially expressed; the red color highlights the compounds whose concentration in glaucoma is significantly different from that in glaucoma. Marked alterations were found in amino acid metabolism, in amino acid-mediated production of secondary metabolites, including secondary amino acid metabolism, in the composition of various membrane lipids, and in the handling/transport of lipids across the mitochondrial membrane. It should be noted that, with the exception of C10 acyl-carnitine, the glaucoma samples showed an increase in the concentration of metabolites whose levels were significantly altered, from amino acids and biogenic amines to lipids and acyl-carnitines. Finally, it was noticed a significant increase (*p* < 0.001) in kynurenine, 0.49 μM in controls to 0.76 μM in glaucoma.

## Discussion

The composition of the AH is important for understanding the physiology of the eye and the homeostatic mechanisms that support the sense of sight. In addition, it is likely that alterations in the concentration of the compounds in the AH provide information on the pathophysiology of diseases that affect the eye and/or the optic nerve.

Here we present novel information, namely the composition of AH sampled from healthy individuals with no known eye disease. Comparing our data with other studies using control samples from controls with cataracts, there are many similarities but also important differences. A limitation of our study is that controls and patients were not matched for age. The reason is that glaucoma appears late in life and therefore the age of the patients was relatively high. In contrast, controls were selected for not having cataracts and this led to the selection of younger individuals.

Table 1 shows that there are 4 of 22 compounds whose concentration is markedly different when comparing our results with those of Buisset et al. (3). Three of them were taurine, butyrylcarnitine, and acetylornithine, all of which we found increased in the glaucoma samples. Although the levels of isoleucine and glutamine were similar in the two studies, the concentration of glycine and alanine was lower in our study and that of leucine was higher in our study. We confirmed the reported increases in lysine and arginine in glaucoma samples compared to controls who underwent cataract surgery (4, 19), but not in glycine as the difference between cases and controls was not significant (Supplementary Table S1). The reasons for these discrepancies do not seem to be due to a different surgical procedure but, probably, to a similar but not equal composition of compounds in the AH of healthy controls and individuals with cataracts.

The composition of samples from glaucoma patients differs from that of controls; in fact, 57 of 80 compounds were increased in the glaucoma samples. 10 compounds had fold changes >2 or <1/2. The level differences for other compounds were neither doubled nor halved, but in some cases the changes in concentration were marked. Apart from glutamine, whose median was 563 in the control vs. 697 μM in the glaucoma individuals, the concentration of kynurenine, lysoPC a 24:0, C12:1 acyl-carnitine and asymmetric dimethylarginine was substantially higher in the glaucoma samples. As noted above, the only compound whose concentration decreased in the glaucoma samples was C10 acyl-carnitine. Regarding spermine, we are aware of the report on variations in glaucoma (3) our study has not provided reliable results for spermine and, therefore, we cannot say whether or not spermine levels vary in glaucoma; on the contrary, it was possible to reliably determine the level of spermidine, which is higher in glaucoma samples. We did not find the decreases in taurine reported elsewhere (3) but we did find increases. The level of taurine in the healthy controls used here is lower than in the study by Buisset et al. (3) and this may be the reason why we detected significant increases in this biogenic amine in glaucoma samples. The reasons for the discrepancies between studies are not readily apparent as the two studies found, in the glaucoma samples, similar increases in the concentration of other compounds. Comparing controls (cataract) and glaucoma samples, the study by Tang et al. (17) did not look for spermine or taurine concentration and its metabolomic profile led to the identification of compounds that were not determined in our study, e.g., purine metabolism intermediates, and of 3-(4-hydroxyphenyl)-propionic acid, N-lactoyl-phenylalanine, D-mannitol, guanidinoethyl sulfonate, hydroxyacetone, 2-aminoadipic acid, cAMP, 3′-sialyllactose, 2-methylbenzoic acid, dulcitol, lysoPC18:0 and lysoPC 15:0.

Principal component analysis of our data clearly separates controls from patients using the 10 selected compounds (Figure 3). The sensitivity and specificity for the 10 selected compounds and for glutamine stand out (Figure 2, Table 2). It should be noted that the increase in the levels of many compounds occurs in the AH of patients who are under therapy to control glaucoma, so the therapy, being useful to prevent optic nerve damage, does not restore the “standard” composition of the humor. Our study confirms the alteration in the level of acyl-carnitines and phosphatidylcholines reported in the Buisset et al. (3) and Tang et al. (17) studies, but increasing the number of compounds whose concentration is altered and better defining the cut-off values for all of them (see Figure 1 and Supplementary Table S1). Of the 5 compounds that were determined in both Tang et al. (17) and in our study, the fold change (glaucoma vs. control) of 4 of them (16:0, 16:1, 18:0 and 18:1 lysophosphatidylcholines) was similar but for 2-aminoadipic acid Tang et al. reports a decrease (log_2_ FC = −0.73) and we report a small increase (log_2_ FC = 0.27). The reason may be the different methodology and/or the fact that in one case the controls had cataracts while in the other only samples from healthy individuals were used.

It is of interest to speculate on the reason for the changes in glutamine, lysophosphatidylcholines, and acyl-carnitines in the AH of glaucoma patients. Increased glutamine levels in glaucoma may be a consequence of trying to keep glutamate (Glu) concentration unchanged. As glutamine is considered generally safe and is used for cryopreservation of tissues/cells (20, 21), an increase in [glutamine] to keep glutamate concentration low (and constant) could be a mechanism to prevent excitotoxic damage caused by accumulation of the amino acid. In paired values in a non-human primate glaucoma model, the concentration of glutamate in the AH before and after the onset of glaucoma was not significantly different. In such a model there was a marked degree of variability (ranges: 2.95–87.3 μM before intervention and 2.77–87.4 after establishment of glaucoma) that we do not find in human samples. In contrast, several years ago, it was reported that elevated intraocular pressure and increased glutamate can lead to the death of retinal ganglion cells. In the study it is interesting the information about glutamate concentration: “*elevated level of glutamate in the vitreous humor of glaucoma patients (27 microM as compared to 11 microM in the control population)*” (22). We wonder if such a high concentration in the vitreous humor of the patients was due to the lack of treatment to normalize ocular pressure; our study shows AH concentrations of 6.55 μM in controls and 7.40 μM in patients with glaucoma.

The altered levels of acyl-carnitines and key membrane phospholipids, suggest that in glaucoma, even if corrected with medication, there are alterations in the membranes, mainly of mitochondrial origin. In fact, carnitines are necessary for the transport of lipids across the mitochondrial membrane. On the one hand, the content of lysophosphatidylcholines is higher in the mitochondrial membranes than in the plasma and endoplasmic reticulum membranes. On the other hand, it is tempting to speculate that the complex phospholipid transport machinery of mitochondrial membranes (23) is altered in glaucoma. Evidence from lymphoblasts from glaucoma patients points to mitochondrial alterations leading to impaired ATP production through defective function of Complex I of the electron transport chain (24, 25); such changes could eventually modify the serum levels of mitochondrial markers that may end up modifying their concentration in the AH. There is also a correlation in increases in 34:2, 34:4 and 36:4 phosphatidylcholines in plasma and AH samples from glaucoma patients (26). However, the involvement of local events affecting the variety of structures that make up the eye cannot be ruled out. A mitochondrial implication may be behind the significant increase in kynurenine in glaucoma samples, since the kynurenine pathway is key in the metabolism of nicotine and adenine dinucleotides, which are essential, among others, for the production of ATP in mitochondria. Targeting the kynurenine pathway has been proposed to combat neurodegeneration caused acutely by hypoxia/ischemia, or chronically due to expression of mutant proteins leading to Huntington's disease or Parkinson's disease (27–29).

Previous studies have shown increases in AH of glaucoma samples of C0, C2, C3, and C4, but to our knowledge, no one has tested (in this body fluid) the other acyl-carnitines that have been determined here. Those studies also show that C4 (butyryl)-carnitine) is found in both the AH and plasma of glaucoma patients (3, 26). In our study, all acyl-carnitines whose concentration is above the detection limit, 13 in total, increased in glaucoma, with two exceptions i) C4:1 and C14:2OH, whose concentrations were similar, and ii) C10, which decreased in the glaucoma samples. These results confirm the mitochondrial alterations and the need to evaluate the role of (decanoyl)-carnitine C10 in the functionality of the mitochondria. This compound showed interest in the past because exogenous administration led to impaired mitochondrial handling and fatty acid oxidation and inhibited ketogenesis (30). Apparently, acyl-carnitines have the potential to better understand the pathophysiological mechanisms of ocular diseases, in the same way that the analysis of the profile of acyl-carnitines has served to better understand monogenic diseases that affect the metabolism of organic acids and of fatty acids (31).

Taken together, the data herein serve to better understand the composition of AH in healthy controls. Due to the power of the approach, which is based on mass spectrometry, amino acids, biogenic amines but also lipophilic molecules such as glycerophospholipids, sphingomyelins and acyl-carnitines could be measured. Glaucoma, even when the ocular pressure is kept under control by pharmacological means, causes a variation in the level of 71% of the measured compounds. The imbalance in the glaucoma samples was substantial for glutamine, probably due to the need to keep the concentration of glutamate low, which is toxic to retinal cells if it accumulates in the AH. The comparison of glaucoma and control samples confirmed that mitochondria play a role in the disease as acyl-carnitines, which are essential for mitochondrial function, were significantly altered.

## Data Availability Statement

Virtually all data obtained from metabolomics analysis are in the Supplementary Material. Any data allegedly missing from the Supplementary Material may be obtained from the corresponding author upon reasonable request.

## Ethics Statement

The study has been evaluated by the Comitè d'Ètica de la Investigació de les Illes Balears (CEI-IB) and deemed not to require ethics approval samples are considered waste and no data on patient identification (neither name, address nor ID numbers) are available to experimenters.

## Author Contributions

RF and JS-N designed the project. JS-N was the surgeon that took the samples. SM and AL did, in single blinded conditions, all experimental and *in silico* work related to processing samples until obtaining raw data. Data analysis and construction of tables and figures was done by JS-M and NB. RF, GN, and MC supervised all the project and validated the final results. MC, JS-N, and RF wrote the first draft of the manuscript. The manuscript was further edited by all authors who approved the submitted version.

## Conflict of Interest

The authors declare that the research was conducted in the absence of any commercial or financial relationships that could be construed as a potential conflict of interest.

## Publisher's Note

All claims expressed in this article are solely those of the authors and do not necessarily represent those of their affiliated organizations, or those of the publisher, the editors and the reviewers. Any product that may be evaluated in this article, or claim that may be made by its manufacturer, is not guaranteed or endorsed by the publisher.
