# Supplementary material for: Targeted Metabolomics Shows That the Level of Glutamine, Kynurenine, Acyl-Carnitines and Lysophosphatidylcholines Is Significantly Increased in the Aqueous Humor of Glaucoma Patients
Source: Front Med (Lausanne). 2022 Jul 22;9:935084. doi: 10.3389/fmed.2022.935084 (PMC9354463; doi:10.3389/fmed.2022.935084)
Supplement: Supplementary file 2 [file Data_Sheet_1.PDF]

Supplementary Material

Targeted metabolomics show that the level of glutamine, acyl-carnitines and lysophosphatidylcholines is significantly increased in the aqueous humor of glaucoma patients.

Authors

Alejandro Lillo*[1,2], Silvia Marin*[3,4,5], Joan Serrano-Marín[6], Nicolas Binetti[6], Gemma Brugal[1,2], Marta Cascante[3,4,5], Juan Sánchez-Navés*[7] and Rafael Franco*[1,6,8]

Supplementary Table S1 is in another file (Excel file)

Supplementary Table S2. Descriptive table of the Eigenvalues and % of variance of each principal component. These data complement the information provided in Figure 3 in the manuscript.

Number of component	***Eigenvalue***	***% of variance***
1	2.50	62.61
2	0.81	20.31
3	0.47	11.20

Supplementary Table S3. The variable coefficients are shown for each principal component formula. These data complement the information provided in Figure 3 in the manuscript and in Supplementary Table S2.

Metabolite	***PC1***	***PC2***	***PC3***
**C3-DC (C4-OH)**	0.37	0.87	-0.23
**Ala**	0.53	-0.46	-0.25
**Ac-Orn**	0.52	0.02	0.85
**Creatinine**	0.56	-0.17	-0.40
